# A loss function to evaluate agricultural decision-making under uncertainty: a case study of soil spectroscopy

**DOI:** 10.1007/s11119-022-09887-2

**Published:** 2022-03-12

**Authors:** T. S. Breure, S. M. Haefele, J. A. Hannam, R. Corstanje, R. Webster, S. Moreno-Rojas, A. E. Milne

**Affiliations:** 1 Cranfield University, Cranfield, Bedfordshire, MK43 0AL UK; 2 Rothamsted Research, Harpenden, AL5 2JQ UK; 3 Gs Growers Ltd, Ely, CB7 5TZ UK

**Keywords:** Geostatistics, Precision agriculture, Variable-rate application, Proximal soil sensing, X-ray fluorescence

## Abstract

Modern sensor technologies can provide detailed information about soil variation which allows for more precise application of fertiliser to minimise environmental harm imposed by agriculture. However, growers should lose neither income nor yield from associated uncertainties of predicted nutrient concentrations and thus one must acknowledge and account for uncertainties. A framework is presented that accounts for the uncertainty and determines the cost–benefit of data on available phosphorus (P) and potassium (K) in the soil determined from sensors. For four fields, the uncertainty associated with variation in soil P and K predicted from sensors was determined. Using published fertiliser dose–yield response curves for a horticultural crop the effect of estimation errors from sensor data on expected financial losses was quantified. The expected losses from optimal precise application were compared with the losses expected from uniform fertiliser application (equivalent to little or no knowledge on soil variation). The asymmetry of the loss function meant that underestimation of P and K generally led to greater losses than the losses from overestimation. This study shows that substantial financial gains can be obtained from sensor-based precise application of P and K fertiliser, with savings of up to £121 ha^−1^ for P and up to £81 ha^−1^ for K, with concurrent environmental benefits due to a reduction of 4–17 kg ha^−1^ applied P fertiliser when compared with uniform application.

**Supplementary Information:**

The online version contains supplementary material available at 10.1007/s11119-022-09887-2.

## Introduction

It is estimated that globally 9–14 million tonnes of phosphorus (P) leaches from fields into watercourses (Beusen et al., 2016; Chen et al., 2016), largely as a result of excessive application of fertiliser. This excessive use of fertiliser is causing substantial environmental harm. To minimise such harm, fertiliser needs to be applied more precisely than at present, varying across fields to meet crop requirements but no more. This accords with the aims of precision agriculture.

Precision agriculture (PA) aims to produce sufficient crops sustainably for society's needs while minimising costs to the producer and harm to the environment. This involves the management of spatial and temporal variation within fields; it requires intense information. Growers who adopt PA must consider the need, value, costs and possible other sources of information to identify whether they can use it to improve their efficiency and reduce environmental impact. The associated uncertainty of the information they acquire for this purpose affects both its value and consequently their view of this approach to management. Current decision-making on variable rate application (VRA) of fertiliser is based primarily on yield responses as functions of management inputs in agronomic trials (Pringle et al., 2004a, 2004b). To vary fertiliser intelligently, however, growers need to have detailed maps of nutrient status or fertiliser requirement for their fields.

Obtaining detailed soil information to support VRA requires intense sampling. Although the required sampling density depends on specific circumstances, in particular the degree of short-distance variation in the soil, a general consensus is that around 10 observations per hectare are required. Both the sampling and laboratory analysis of soil collected are laborious and time-consuming. The whole process by conventional wet chemical analysis is too expensive for the sizes of samples required to map soil variation accurately within fields. Most growers therefore apply uniform dressings of fertiliser based on average estimates of nutrient concentrations made from a small number of samples. Recent developments in spectroscopy offer an affordable and effective alternative with instruments designed for use both in the laboratory and in the field (Li et al., 2015). X-ray fluorescence (XRF) spectroscopy has been available for several decades, and more recently infrared (IR) reflectance spectroscopy has become feasible for analysing and predicting soil properties on a large number of samples cheaply (Bellon-Maurel & McBratney, 2011; Viscarra Rossel & Webster, 2012). The net result is substantially cheaper than chemical analysis alone and enables surveyors to obtain sufficient detail on the spatial variation of soil properties affordably (Viscarra Rossel & Bouma, 2016).

All measurement embodies some degree of error, however, and so soil data also have their associated uncertainty. Nevertheless this study considered the errors in standard chemical analyses by modern equipment small enough to be ignored. Spectroscopic estimation introduces yet another source of error. That is, calibration equations that describe the relationship between the wet chemistry measures and the soil spectra also have an associated error. Finally, measurements of the soil cannot be made at all locations, and so interpolation is necessary to estimate the soil variables between measured locations. This interpolation, usually done by kriging, has an associated prediction error. These errors accumulate through the whole procedure and are embodied in the error variances of the final estimates. Ignoring the resultant uncertainty can lead to false inferences from the data and hence faulty decision-making (Cherry et al., 2008; Goovaerts, 2001; Heuvelink, 2018).

Given the ease with which spectroscopy can replace conventional chemical methods for analysing soil, it is important to know how the errors it introduces affect the final spatial predictions and in turn the potential financial advantage of precise fertiliser management compared with uniform application. This is because errors carry with them costs. Over-estimation of a plant nutrient concentration in the soil, say P, would lead to a grower's applying too little fertiliser and to loss of potential yield and income. Under-estimation of the concentration would lead to the grower's over-fertilizing, spending unnecessarily on fertiliser, to the point of spending more than earned in increased yield of crop.

Yates (1981) set out the principles by which one might assess the balance between costs of survey and benefits that would accrue from greater accuracy. The aim is to minimise the sum of sampling costs and expected losses due to errors. For this Yates defined a loss function and suggested how it might be minimised. The loss function is a generic approach studied not only in the context of soil survey (Lark & Knights, 2015) but also in environmental protection (Goovaerts, 1997) and soil management (Faechner et al., 2000). Because overestimation and underestimation incur losses for different reasons the loss function may be asymmetric. Given a loss function and an error distribution for the information, one can make a decision that minimises expected losses (e.g. Faechner et al., 2000; Goovaerts, 1997).

This study is concerned with the prediction of available P and K for precise management of fertiliser in horticultural crops. The main objective is to investigate the potential for soil spectral methods (near- and mid-infrared and X-ray fluorescence) to predict how these soil variables change within well-managed fields and so determine the effect of prediction errors on the expected loss. It was hypothesised that, once uncertainty in predictions has been accounted for, soil spectroscopy could adequately predict the spatial variability to justify variable rate application of P and K fertiliser. For this, soil samples from four fields in the Cambridgeshire Fens of the UK were considered. Measurements of available P and K estimated from soil spectra were used to predict how the concentrations vary across the fields and to compute the associated error variances from interpolation. The expected losses associated with varying applications of fertiliser given the error variance of the predictions are computed. The expected losses are compared with the losses that would have accrued if estimates from wet chemistry had been used to determine a single application rate per field. General conclusions are drawn about the effect of uncertainty in nutrient status on economic and environmental losses and practical considerations are given with regard to implementing the loss function.

## Methods

### Data

Data were obtained in sample surveys of four fields in the Fen district of Cambridgeshire, England, in 2018 and 2019. The region was originally dominated by peat, much of which has oxidized since the land was drained in the seventeenth century. The land surface is now 1 to 2 m lower than it was except for the natural sinuous drainage channels containing mineral sediment. These former channels, known locally as ‘rodhams’, have become elevated features in the landscape (Hodge et al., 1984), and they are clearly distinguishable on LiDAR (light detection and ranging) imagery. The LiDAR raster (2 m × 2 m resolution) from the British Environment Agency was used as a basis for sampling. The sampling design of Field 1 (8.2 ha) was based around a 30-m square grid, with three transects (on alternate rows of the grid) more intensely sampled at 6-m intervals. The designs for Field 2 (16.9 ha), Field 3 (5.1 ha) and Field 4 (8.9 ha) were computed for initial numbers of points (121, 107 and 100, respectively) by spatial coverage sampling (Walvoort et al., 2010). Each point lay in the centre of its Dirichlet tile. All tiles in each field were of equal area, ensuring spatial coverage of the entire field. This led to an approximate grid with an interval of around 30 m. A sub-sample of 36 (Field 2), 26 (Field 3) and 32 (Field 4) of these points were selected with balanced sampling (Grafström & Lilics, 2019) on the spatial coordinates and LiDAR. At each location of these sub-samples, another sampling point 6 m away at a random orientation was added to estimate the short-scale spatial variance. In all fields extra sample points were also added to ensure coverage of the range of soil conditions and LiDAR. The decision for the location of these extra points was based on the LiDAR survey and satellite imagery showing variation in soil colour. In all, the numbers of sampling points for the fields were 256 (Field 1), 161 (Field 2), 138 (Field 3) and 142 (Field 4). Supplementary Fig. 1 shows the field boundaries with the sampling points. Three soil cores of topsoil (0–0.25 m) were taken within a 0.5 m × 0.5 m quadrat at each sampling location. These three cores were bulked and mixed for spectroscopic measurements. A subset of 30 samples from each field was taken for further laboratory analysis. The subset was selected from the total sample by balanced sampling on the spatial coordinates and LiDAR data.

Available P was measured by the standard Olsen method (Olsen et al., 1954) and a SANplus continuous colorimetric flow analysis (Skalar Analytical BV, Breda, Netherlands). Available K was determined in an ammonium-nitrate (NH_4_NO_3_) extract and Inductively Coupled Optical-Emission Spectroscopy (Optima 7300 DV, Seer Green, UK). The soil samples were dried in air and milled, and sub-samples were pressed into small wells (6 mm across and approximately 1 mm deep) and placed in a Tensor II spectrometer (Bruker, Ettlingen, Germany). The absorbance spectrum in the range 9998–3999 cm^−1^, i.e. the near-infrared (NIR), of each sub-sample was measured with a resolution of 1 cm. Each sub-sample's mid-infrared (MIR) spectrum in the range 4000–600 cm^−1^ was recorded on the same instrument with a resolution of 2 cm^−1^. A DP-6000 Delta Premium portable X-ray fluorescence (pXRF) (Olympus Ltd, Center Valley, USA) was used to scan the soil samples. The pXRF features a Rh X-ray tube operated at 10–40 keV with a high resolution ($<$ 165 eV) silicon drift detector. The pXRF was set to scan for 30 s at both 10 and 40 keV. The pXRF was set up in an instrument stand, and samples where placed on the aperture in a sample cup covered with a Prolene Thin Film (Chemplex Ind, Florida, USA). Potential drift in the XRF analyser was reduced by scans of a stainless steel 316-alloy clip containing 16.13% Cr, 1.78% Mn, 68.76% Fe, 10.42% Ni, 0.20% Cu, and 2.10% Mo tightly fitted over the aperture prior to the measurements on each aliquot. The pXRF samples where measured in three replicates on one aliquot, near- and mid-infrared spectra were measured on three aliquots of each soil sample. Further analysis was done on the mean spectra of those three measurements.

### Spectral processing and calibration

The raw spectra were pre-processed first by the Savitzky–Golay filter (Savitzky & Golay, 1964) and then transformed to their first derivatives. The H_2_O bands (7900–5587 and 6849–5102 cm^−1^) were removed from the NIR spectra (Bowers & Hanks, 1964). The region of 4464–4115 cm^−1^ was removed from the MIR spectra to account for the CO_2_ peak at 4248 cm^−1^ (Sandford & Allamandola, 1990). The 10 keV XRF spectra were subset to the range of 0.5–7.8 keV, the 40 keV XRF spectra to the range of 0–24.4 keV. The 10- and 40-keV spectra were then combined.

Calibration was done by partial least squares (PLS) regression with the kernel algorithm on the derivative spectra. Separate models were used for Fields 3 and 4, whereas a single model was used on pooled data for Fields 1 and 2 because they were close to one another. The number of components to be included in the model were selected as follows. First, the mean squared error (MSE) between the known values and the predictions was computed by leave-one-out cross-validation (LOO-CV). The standard deviation of the LOO-CV residuals was also computed. To minimise over-fitting, models were computed for a maximum of 15 components. Subsequently, the model was chosen that included the fewest components, yet lay within the MSE's standard deviation of the model that had the smallest error overall (Hastie et al., 2009, Sect. 7.10). The minimum number of components to be included was set to 1.

Preliminary analysis showed that whilst XRF tends to give the most accurate predictions of P and K, this was not always the case, and therefore all three sets of spectra were combined. The PLS predictions from NIR, MIR and XRF matrices for each property were used for an ordinary least-squares (OLS) multiple regression, known as the Granger–Ramanathan averaging method (Granger & Ramanathan, 1984). The model underlying the OLS regression in its general form is

1 $${\mathbf{y}}_{\text{chem}} = {w}_{0}+{w}_{1}{\mathbf{z}}_{1}+\cdots +{w}_{t}{\mathbf{z}}_{t}+{\varvec{\upvarepsilon}}$$

where ${\mathbf{y}}_{\text{chem}}$ is a vector of observed values (as measured by wet chemistry), $\mathbf{z}$ is a vector of PLS predictions, the ${w}_{i}, i=\mathrm{1,2},\dots ,t$, are weighting coefficients of the $t$ individual predictors included in the regression and ${\varvec{\upvarepsilon}}$ is the error term. This equation was solved for the intercept (${w}_{0}$) and the $t$ coefficients for each of the spectral matrix combinations ($\mathbf{z}$). The intercepts correct for bias if one of the individual predictors is biased.

Each prediction has associated with it an error, and these errors were treated as ones arising from the use of the OLS regression model. The error variance of these predictions, of which there are $n$, is hereafter referred to as

2 $${\text{var}}\left[{\varepsilon }_{y}\right] = \frac{1}{n}{\sum }_{i=1}^{n}{\left\{{y}_{\text{chem}}\left(i\right)-\widehat{{y}_{\text{spec}}}\left(i\right)\right\}}^{2}$$

where ${y}_{\text{chem}}\left(i\right)$ are the assumed true values measured by wet chemistry and $\widehat{{y}_{\text{spec}}}\left(i\right)$ are the predicted values from the spectra. The error variance has been propagated through into the geostatistical model. The reader is referred to Supplementary Fig. 2 for further details on the PLS and Granger–Ramanathan model-averaging results.

### Geostatistical analysis

The predictions from the spectra together with the spatial coordinates and LiDAR heights were then used to predict the concentrations of each nutrient by the empirical best linear unbiased predictor (E-BLUP) (Lark et al., 2006). This is effectively a combination of kriging the spectral estimates with external drift (the LiDAR height) and universal kriging. It combines additively fixed effects (e.g. the unknown mean, the LiDAR height and the coefficients of any trend) and random effects (the spatially correlated random variation). Its model is

3 $${Y}_{\text{krig}}\left(\mathbf{x}\right) = \alpha H\left(\mathbf{x}\right)+{\sum }_{j=0}^{J}{\beta }_{j}{f}_{j}\left(\mathbf{x}\right)+\varepsilon \left(\mathbf{x}\right)$$

Here $\alpha$ is the coefficient of the height, $H$, measured by LiDAR, the $\beta$ are unknown coefficients, $\mathbf{x}\equiv {x}_{1},{x}_{2}$ are the coordinates of a position in the field, the ${f}_{j}\left(\mathbf{x}\right)$ are typically first- or second-order polynomials, and the $\varepsilon \left(\mathbf{x}\right)$ represents the residuals from the fixed effects, i.e. the LiDAR height and the spatial trend. The residuals are assumed to be second-order stationary random variables, jointly normally distributed with zero means and $n\times n$ covariance matrix ${\mathbf{C}}_{\text{d}}$ with variogram $\gamma \left(\mathbf{h}\right)$:

4 $$\gamma \left(\mathbf{h}\right)=\frac{1}{2}{\text{E}}\left[{\left\{\varepsilon \left(\mathbf{x}\right)-\varepsilon \left(\mathbf{x}+\mathbf{h}\right)\right\}}^{2}\right]$$

where $\mathbf{h}$ is the lag in distance and direction between any two points. The random variation was treated as isotropic, so that $\mathbf{h}$ becomes a scalar in distance only: $h=\left|\mathbf{h}\right|$. The variogram of $\varepsilon \left(\mathbf{x}\right)$ was examined by the method of moments. In all cases the random residuals could be successfully described by the isotropic exponential variogram model:

5 $$\begin{aligned}\gamma \left(h\right)&={c}_{0}+{c}_{1}\left\{1-\mathrm{exp}\left(-\frac{h}{a}\right)\right\}\,{\text{for}}\,0<h \\ &=0 \,{\text{ for }}\,h=0\end{aligned}$$

or the isotropic spherical variogram model:

6 $$\begin{aligned}\gamma \left(h\right)&={c}_{0}+{c}_{1}\left\{\frac{3h}{2r}-\frac{1}{2}{\left(\frac{h}{r}\right)}^{3}\right\}\,{\text{for }}\, 0<h<r \\ &={c}_{0}+{c}_{1}\,\text{ for }\,h\ge r \\&=0 {\text{ for }} h=0 \end{aligned}$$

Here ${c}_{0}$ and ${c}_{1}$ are variances, respectively the nugget and sill of the correlated variance, and $r$ (the range of the spherical function) and $a$ are distance parameters. Parameters for a plausible model can be found by maximum likelihood (ml) or maximization of the likelihood of the residuals given the data (reml). Lark et al. (2006) and Webster and Oliver (2007) give the derivation of the equations in full. The reml estimation method is preferred, because it reduces bias in random effects parameters due to the uncertainty in the fixed effects parameters. However, the ml may be compared between models with different fixed effects structures, but such a comparison is not valid for reml. Therefore, the ml method was used first to select the fixed effects.

Preliminary investigations (visual inspection and marginal plots) suggested that the estimated soil properties, $\widehat{{y}_{\text{spec}}}$, have long-range trends across the fields. These were considered as fixed effects. Estimated soil properties also vary systematically with elevation as recorded by LiDAR, so this was considered as another fixed effect. Each trend variable (up to quadratic terms in eastings and northings, and elevation) was added in turn and tested whether its addition was significant by a log-likelihood ratio test. A chi-squared $p$-value from the log-likelihood ratio of 0.05 was taken as threshold and any smaller value ($p \le 0.05$) was treated as evidence that additional trend parameters should be included. After the selection of fixed effects, the fixed effects and random effects in Eq. (3) were re-estimated using residual maximum likelihood (reml). If ${c}_{0}$ was less than ${\text{var}}\left[{\varepsilon }_{y}\right]$ then it was set equal to ${\text{var}}\left[{\varepsilon }_{y}\right]$ and Eq. (3) was solved again. Next, the final variograms were used for universal kriging. This provided the predictions and their kriging variances.

The linear mixed models were cross-validated by LOO-CV. The linear mixed models were re-estimated for each iteration to diminish bias in parameter values (Hastie et al., 2009, Sect. 7.10). The LOO-CV of the linear mixed-models were evaluated with the mean- and median-standardized squared prediction errors (SSPEs) (Lark, 2000).

### Theory of the loss function

The loss function, $L\left(F\right)$, is defined as the difference in profit that results from applying a given amount of fertiliser $F$ compared with the economic optimal amount ${F}_{0}$:

7 $$L\left(F\right) =\Phi \left({F}_{0}\right)-\Phi \left(F\right)$$

where the profit $\Phi \left(F\right)$ is the difference between the income from the crop (price of the crop × yield) and the cost of the fertiliser:

8 $$\Phi \left(F\right) = M\times {\text{Yield}}-V\times F$$

where $M$ is the price of the crop (£ t^−1^) and $V$ is the cost of the fertiliser (£ kg^−1^). It was assumed that the yield is given by the dose–response equation:

9 $$\psi = \alpha +\eta {R}^{\xi F+S}+\upnu \left(\xi F+S\right)$$

where $\psi$ is the yield, $S$ is the concentration of the nutrient in the soil, $F$ is the applied fertiliser (kg ha^−1^), $\xi$ is the increase in nutrient concentration (mg kg^−1^) in the soil for every 1 kg ha^−1^ fertiliser applied, and $\alpha$, $\eta$, $\nu$ and $R$ are parameters, then the optimum amount of fertiliser can be calculated from this and is given by

10 $${F}_{0} =\mathrm{ ln }\left(\frac{B / \xi - \nu }{\eta {R}^{S} \mathrm{ln} R }\right)/ \xi ln R$$

where $B=V/M$, known as the break-even ratio.

By definition, the loss given by Eq. (7) is zero when the optimum amount of fertiliser is applied. However, computing the optimum amount of fertiliser to apply relies on one's knowing the nutrient status, $S$, in the soil, and one cannot know it precisely.

Given the probability distribution, $g\left(s\right)$, of the nutrient status $S$ the optimum fertiliser rate that maximizes the expected profit can be computed:

11 $${F}_{\text{opt}} =\mathrm{ ln}\left(\frac{B/\xi -\nu }{\eta \mathrm{ln}R{\int }_{0}^{\infty }{R}^{s}g\left(s\right){\text{d}}s}\right)/\xi \mathrm{ln}R$$

This also minimises the expected loss function, ${\text{E}}\left[L\left(F\right)\right]$, which was defined here as the difference between the profit where $S$ is known without error and associated optimum fertiliser, ${F}_{0}$ in Eq. (10), is applied

12 $$\mathrm{E}\left[L\left(F,S\right)\right]=\Phi \left({F}_{0}, S\right)- {\int }_{0}^{\infty }\left\{\Phi \left(F,s\right)\right\} g\left(s\right) \text{d}s$$

### Parameterization and analysis of the loss function

All fields sampled were cultivated for lettuce, and so loss functions were computed associated with this crop. The dose response curve for P was derived from Prasad et al. (1988) and for K from Greenwood et al. (1980). Parameters were estimated for the linear plus exponential functions with the Gauss–Newton algorithm in GenStat (VSN International, 2021). It was assumed that for every 1 kg of P added in fertiliser 0.18 kg becomes available to the crop (Muhammed et al., 2017), for every 1 kg of K added in fertiliser, 0.62 kg becomes available to the crop (Blake et al., 1999). Furthermore, it was assumed that the added nutrients are contained in the top 0.25 m of the soil (the sampling depth). The value of 480 kg m^−3^ for bulk density of this peat soil was taken from Milne et al. (2006). Given the support of the kriged predictions (2 m × 2 m), it follows that an addition of 1 kg fertiliser per ha leads to an increase in the concentration of this layer of 0.15 mg available P kg^−1^ and 0.52 mg available K kg^−1^, equal to $\xi$ in the dose–response Eq. (9) (see details in Supplementary Material). Greenwood et al. (1980) listed a mean base nutrient concentration of 69 mg available K kg^−1^ for the unfertilised soil in their study, which was used as an additive component. A profit margin ($M$) of £90 per tonne of lettuce per hectare was assumed. The prices of fertiliser ($V$) were taken as £0.36 per kg P fertiliser and £0.29 per kg K fertiliser. Table 1 lists the parameter values of the dose–response equations for P and K.

**Table 1:** Dose—response equation parameters as relevant to Eq. 9

Soil nutrient	$\alpha$	$\eta$	$\nu$	$R$	$\xi$
P	142.15	− 145.8	− 0.776	0.98	0.15
K	63.3	− 63.3	0	0.98	0.52

The profitability of variable-rate application (VRA) was assessed based on kriged maps by computing the total expected loss (Eq. 12) across each field. This was done by comparing the expected loss for each field between that from VRA, ${\text{E}}\left[L\left({F}_{\text{opt}}\right]\right)$, and a uniform application (UA) based on wet chemistry alone, ${\text{E}}\left[L\left({F}_{\text{UA}}\right]\right)$. For this purpose, five samples per field were selected in a W-shape from the locations at which the nutrients had been measured by wet chemistry. These five samples were used to compute an average soil nutrient concentration per field that was used to determine the uniform rate of fertiliser application.

### Software

Analysis was done with base R commands as well as the following R packages as implemented in RStudio: data handling with the **tidyverse** (Wickham et al., 2019) package, computation of the sampling designs using the **spcosa** (Walvoort et al., 2010), **BalancedSampling** (Grafström & Lilics, 2019) and **SpatialEco** (Evans, 2019) packages, spectral processing using **prospectr** (Stevens & Ramirez-Lopez, 2013), partial least squares regression using **pls** (Bjørn-Helge et al., 2019), Granger–Ramanathan averaging using **GeomComb** (Weiss & Roetzer, 2016), model-based geostatistics using the **geoR** package (Ribeiro & Diggle, 2018) and handling of spatial objects using the **raster** (Hijmans, 2020) and **rgdal** (Bivand et al., 2020) packages. Graphics were created with base R functions and the package **ggplot2** (Wickham, 2016).

## Results

### Uncertainty in kriging predictions from soil properties estimated by spectroscopy

**Fig. 1﻿:**
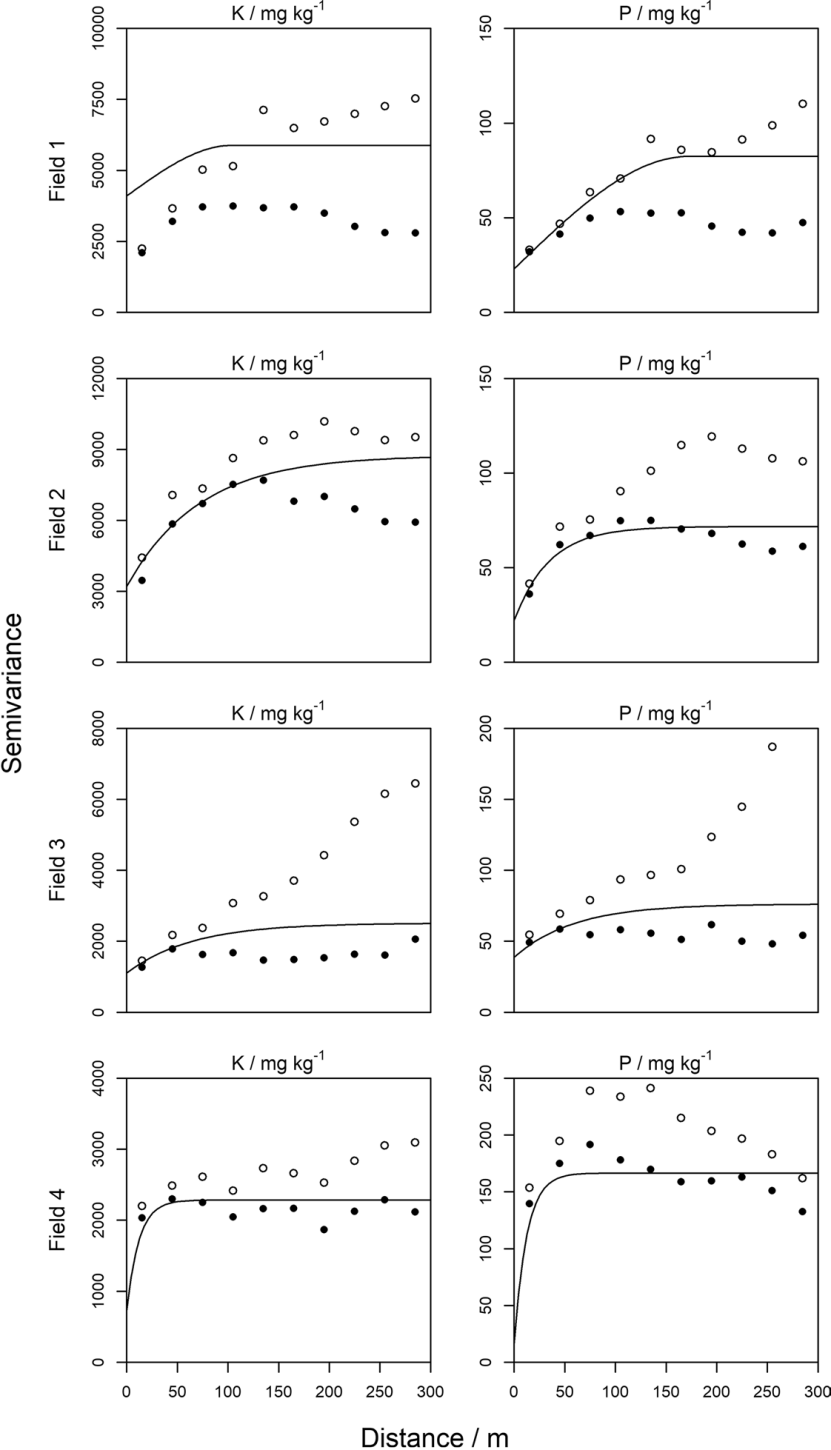
Linear mixed model variograms for all fields, $\circ$ refer to the experimental variograms of the original variable, $\bullet$ refer to the experimental variograms of the residuals from the trend model, the solid black line refers to the final model fitted by restricted maximum likelihood procedures

The nugget variances, ${c}_{0}$, were underestimated for the following variogram models: available K (Fields 1, 2, 3, and 4) and available P (Fields 1 and 2). The nugget parameter was therefore set equal to ${\text{var}}\left[{\varepsilon }_{y}\right]$ and Eq. (3) was solved again to account for the under-estimation of the error. Data of both P and K in all four fields were fitted with a linear trend model as fixed effects (Table 2). Fitting trend coefficients, as expected, resulted in smaller semivariances than their equivalents of the original variables, i.e. the difference between black discs and circles (Fig. 1). The LOO-CV results of the mixed model variograms accorded overall with expectations for both P and K and all four fields (Supplementary Fig. 3).

**Table 2:** Fixed effects and parameters estimated by reml of the variograms, $H$ stands for LiDAR (elevation height in metres), and ${x}_{1}$ and ${x}_{2}$ are the spatial coordinates

Field	Soil nutrient	Fixed effects	Variogram type	Variogram parameters			
				${c}_{0}$	${c}_{1}$	$r / m$	$a / m$
1	P	${x}_{1}, {x}_{2}, {x}_{1}^{2}, {x}_{2}^{2}, {x}_{1}{x}_{2}$	Sph	23	59	172	–
	K	$H, {x}_{1}, {x}_{2}, {x}_{1}^{2}, {x}_{2}^{2}, {x}_{1}{x}_{2}$	Sph	4100	1784	104	–
2	P	$H, {x}_{1}, {x}_{2}$	Exp	22	50	–	35
	K	$H, {x}_{1}, {x}_{2}$	Exp	3200	5551	–	64
3	P	$H, {x}_{1}, {x}_{2}, {x}_{1}^{2}, {x}_{2}^{2}, {x}_{1}{x}_{2}$	Exp	39	38	–	59
	K	${x}_{1}, {x}_{2}, {x}_{1}^{2}, {x}_{2}^{2}, {x}_{1}{x}_{2}$	Exp	1100	1413	–	64
4	P	$H$	Exp	15	151	–	12
	K	$H, {x}_{1}, {x}_{2}, {x}_{1}^{2}, {x}_{2}^{2}, {x}_{1}{x}_{2}$	Exp	720	1565	–	12

The variogram parameters are the nugget variance (${c}_{0}$) the sill of the correlated variance (${c}_{1}$), and $r$ and $a$ are the distance parameters

*Sph* spherical, *Exp* exponential

### Loss function on variable-rate fertiliser application

**Fig. 2:**
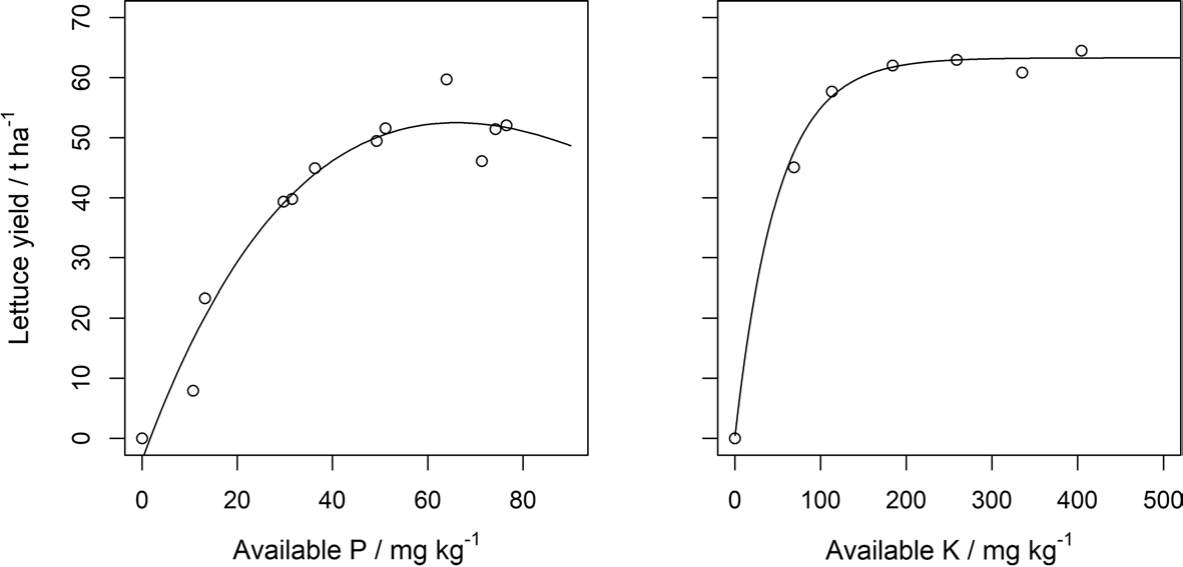
Dose–response curves for available P (exponential + linear) and available K (exponential) fitted based on data from Prasad et al. (1988) (P) and Greenwood et al. (1980) (K). See Table 1 in the main text for parameter values

The fitted dose–response curves for P and K affected the profit $\Phi \left(F\right)$ and the loss function $L\left(F\right)=\Phi \left({F}_{0}\right)-\Phi \left(F\right)$ differently because of their asymmetry characteristics and by association the expected loss, i.e. loss from perfect knowledge, ${\text{E}}\left[L\left(F\right)\right]$ in Eq. (12). For available P, the dose–response curve declines linearly in yield for large values of P (Fig. 2).

**Fig. 3:**
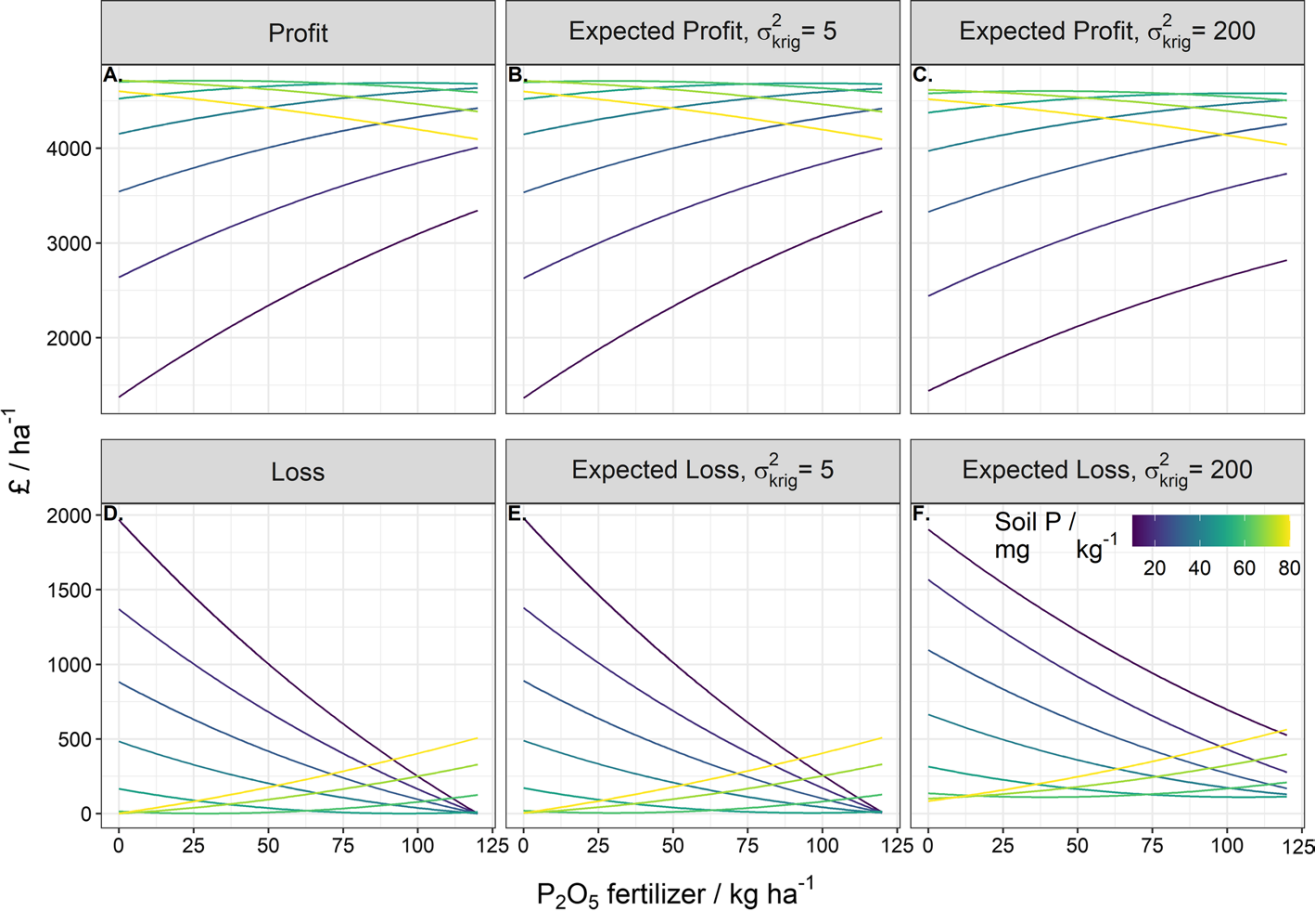
Profit and loss under zero error variance, expected profit and loss under an error variance of 5 mg kg^−1^ and an error variance of 200 mg kg^−1^ for a range of estimated soil P values from 10 to 80 mg kg^−1^. The range of P fertiliser applied spans 0 to 120 kg ha^−1^

**Fig. 4:**
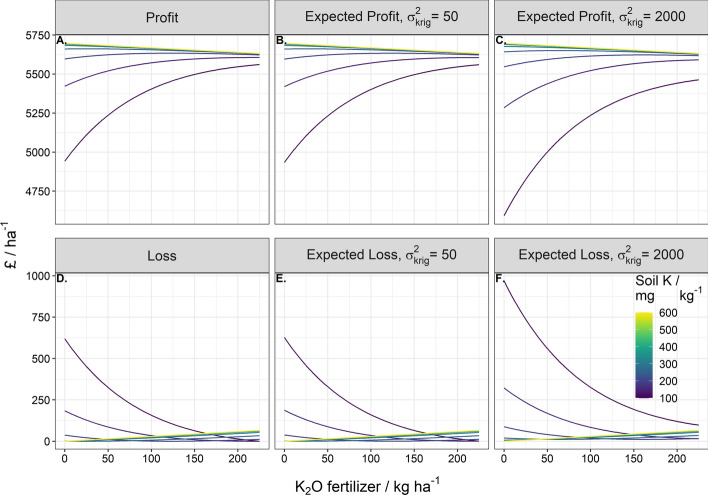
Profit and loss under zero error variance, expected profit and loss under an error variance of 50 mg kg^−1^ and an error variance of 2000 mg kg^−1^ for a range of estimated soil K values from 100 to 600 mg kg^−1^. The range of K fertiliser applied spans 0 to 225 kg ha^−1^

That is to say over-application of fertiliser results in financial losses because too much fertiliser is applied and yield is diminished. This effect of over-application of P fertiliser to soil with a large concentration of P fertiliser is greater than a similar over-application of K fertiliser; for K where the dose–response curve increases to an asymptote, and so the associated prediction of profit and loss converge at large rates of applied fertiliser (Figs. 3 and 4). For both P and K, larger uncertainty of the predicted nutrient content (large ${\sigma }_{\text{krig}}^{2}$) increases the expected loss and reduces the profit.

**Fig. 5:**
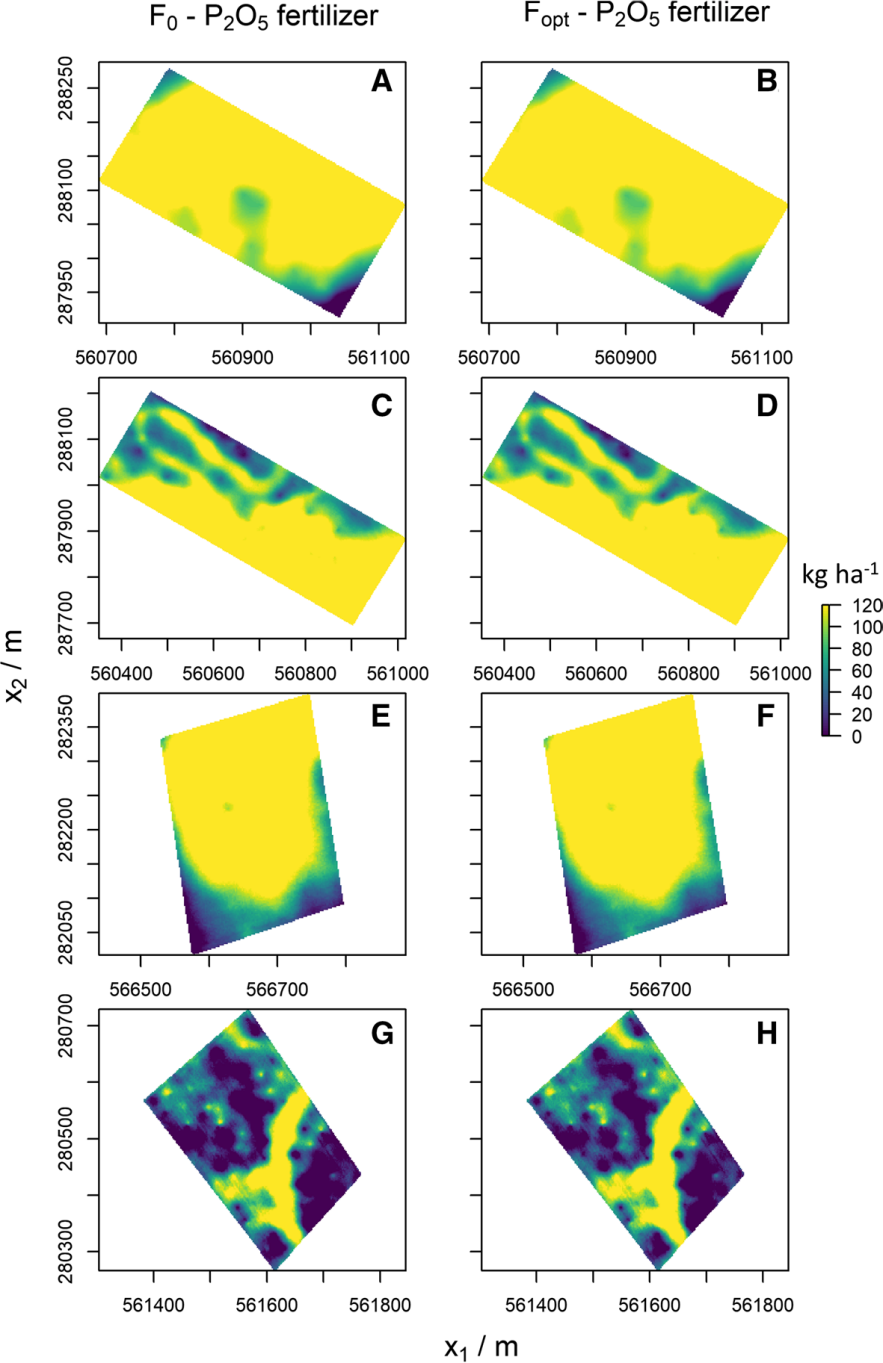
Optimum P fertiliser application with perfect knowledge of soil P (${\mathrm{F}}_{0}$), optimum application when accounting for ${\sigma }_{\text{krig}}^{2}$ in the estimate of soil P (${\mathrm{F}}_{\text{opt}}$)

As expected, the fertiliser rate that minimises the expected loss (${F}_{\text{opt}}$) is greater than the optimal fertiliser rate when the soil variable is known without error (${F}_{0}$) for all fields, again because of the asymmetry of the loss functions. The asymmetry means that overestimation generally leads to larger losses than does underestimation of soil P and K. Error variance in the estimates of P and K in the soil consequently leads to larger recommended applications of fertiliser than if one had perfect knowledge (${\sigma }_{\text{krig}}^{2}=0$) (Fig. 5).

**Fig. 6:**
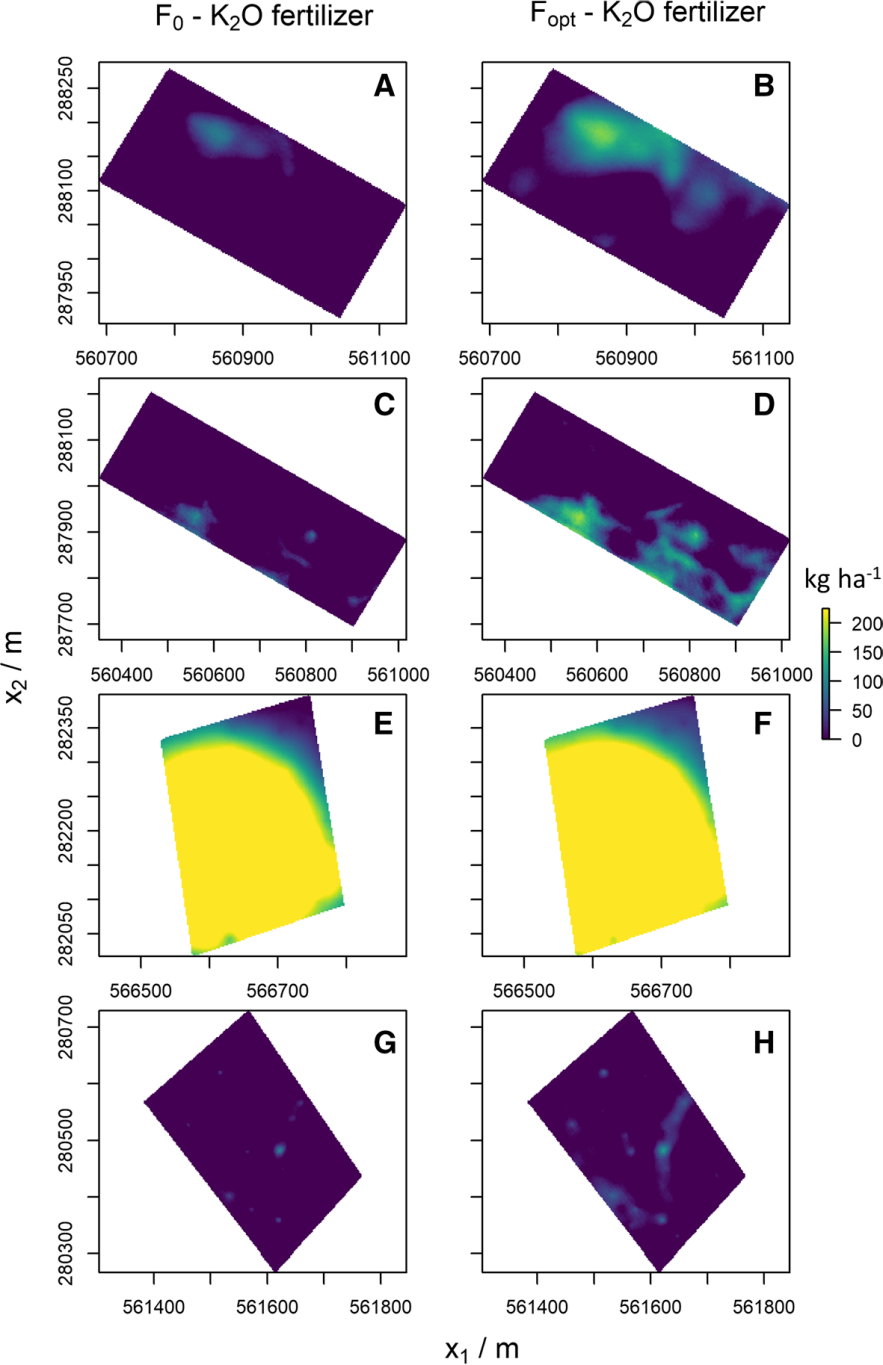
Optimum K fertiliser application with perfect knowledge of soil K (${F}_{0}$), optimum application when accounting for ${\sigma }_{\text{krig}}^{2}$ in the estimate of soil K (${F}_{\mathrm{opt}}$)

**Fig. 7:**
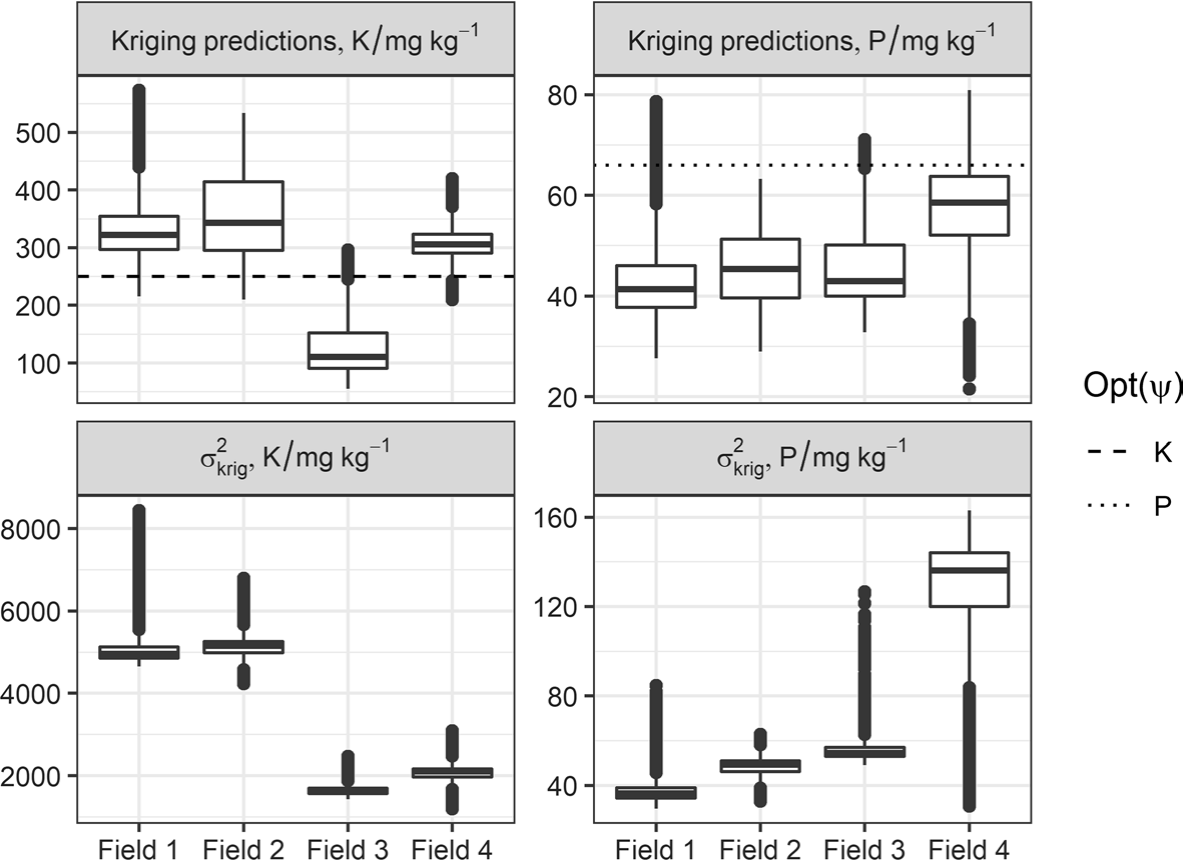
Box-plots of kriging predictions and the kriging variance (${\sigma }_{\text{krig}}^{2}$), by field for available P and K, horizontal lines represent the nutrient value for which the maximum yield, Opt($\psi$), is obtained on the fitted dose–response curve

The optimal P fertiliser application varied substantially in all fields. The variation was less pronounced for K, particularly in Field 4. Across Field 1, most kriged estimates of K fall on the asymptote of the dose–response curve, hence ${F}_{0}=0$ for a large part of the field (Fig. 6A). The ${\sigma }_{\text{krig}}^{2}$ increases the probability that kriging estimates fall below the asymptote, however. In those situations application of fertiliser becomes necessary to minimise the expected loss (Fig. 6B). These observations also hold for Fields 2 and 4. The kriged estimates of available K in Fields 1, 2 and 4 were larger than the range of the dose–response curve (Fig. 7). Consequently, applying no fertiliser for a major portion of the field was more profitable (Fig. 6B, D and H).

The total expected loss on a field-basis was less for variable-rate P and K application (${\text{E}}\left[L\left({F}_{\text{opt}}\right)\right]$) than the total loss of blanket fertiliser application arising from the wet chemical analysis (${\text{E}}\left[L\left({F}_{\text{UA}}\right)\right]$) (Table 3). There was a financial incentive of VRA of P fertiliser across all fields (ranging from £7–£121 ha^−1^) and for K fertiliser across three fields (ranging from £6–£81 ha^−1^). That is, for available K the difference between ${\text{E}}\left[L\left({F}_{\text{opt}}\right)\right]$ and ${\text{E}}\left[L\left({F}_{\text{UA}}\right)\right]$ was small in Field 4. Less P fertiliser was used on a field-basis under VRA (${F}_{\text{opt}}$) than with uniform application (${F}_{\text{UA}}$) in Fields 1, 2 and 3 (Table 3). Within those fields, total P fertiliser use was reduced by VRA with 4–17 kg ha^−1^ compared with uniform application. Most K fertiliser would be used on a field-basis in all cases under VRA (${F}_{\text{opt}}$) (Table 3).

**Table 3:** Fertiliser used (kg ha^−1^) for perfect knowledge (${F}_{0}$), variable-rate application (${F}_{opt}$) and uniform application based on wet chemistry samples (${F}_{UA}$)

Field	Area (ha)	Soil nutrient	Fertiliser (kg ha^−1^)			Expected loss (£ ha^−1^)		
			${F}_{0}$	${F}_{opt}$	${F}_{UA}$	$E[L({F}_{opt})]$	$E[L({F}_{UA})]$	$\Delta E[L(F)]$
1	8.2	P	113	113	120	31	38	7
2	16.9		102	103	120	38	52	14
3	5.1		103	104	61	33	154	121
4	8.9		47	53	57	94	145	51
1	8.2	K	4	27	0	16	23	7
2	16.9		3	24	0	13	19	6
3	5.1		197	205	144	69	150	81
4	8.9		0	4	0	6	7	1

Expected loss (from perfect knowledge) for variable-rate application﻿, $E[L({F}_{opt})]$, and uniform application, $E[L({F}_{UA})]$, with $\Delta E[L(F)]$ being the difference in expected loss given by $E[L({F}_{UA})]-E[L({F}_{opt})]$

## Discussion

### Error approximation and the estimation of the variogram

For this study it was assumed that the error was homogeneous across the sample locations that were predicted by spectroscopy. This allowed for the more generic assumption that the prediction library already existed (for example, derived from a spectral library). If, however, predictions were obtained from a single sampling campaign, the error variance of locations with associated wet chemistry data and those without could be separated in a subsequent geostatistical analysis, as Delhomme (1978) suggested.

Error propagation is rarely taken into account in soil surveys based on spectroscopy. Ramirez-Lopez et al. (2019), listed two other studies in which the propagation of errors was reported in the last 10 years (Brodský et al., 2013; Viscarra Rossel et al., 2016). Somarathna et al. (2018) and Ellinger et al. (2019) also propagated errors from infrared spectral into predictions of soil carbon. Error propagation is important for two reasons. First, Somarathna et al. (2018) found that acknowledging the measurement error, in this case ${\text{var}}\left[{\varepsilon }_{y}\right]$, reduces uncertainty in spatial predictions (as supported by Clark et al., 2010). The extent to which this has an effect will depend on the complexity of the target variable's spatial variation and the geographical extent of the study. Second, acknowledgement of the uncertainty (and its minimisation) is necessary to detect small rates of change in the soil property of interest by monitoring over time (Viscarra Rossel et al., 2016). This study is concerned with variation in space, and it is this variation that determines whether variable rate application is relevant.

One merit of kriging is that in addition to providing unbiased predictions it minimises the squared-errors of those predictions, which are known. The predictions are best in that sense. Kriging, like other forms of regression, smooths: unobserved small values are over-estimated, and large ones under-estimated (Webster & Oliver, 2007). Smoothing thus leads to underestimation of the spatial variance, a consequence one needs to consider in using the predictions for determining variable rates of fertiliser.

LiDAR was included as a fixed effect in six of the eight LMMs, and all LMMs except one included geographic trends (i.e. in the spatial coordinates) in the model. Kriging within reml is based in the assumption of second-order stationarity of the random part of the process. That is why the fixed effects of trend and LiDAR were separated out and also why each field was treated separately. Having an exhaustive covariate allowed one to do that and to approximate the uncertainty of the target variable more accurately than otherwise (Lark, 2009).

### Data requirements and estimation of the loss function

The loss function requires certain requirements of, or assumptions about, data. For example, the soil's bulk density was estimated from general knowledge in the area (see method Sect. 3.4). It is known that the density of soil on the rodhams differs from that of the peaty soil between them. Even in the best scenario, these estimates embody an error which should ideally be accounted for. Similarly, the modelled response of the crop contains error and this should be incorporated in the framework, although this was not included for reasons of clarity.

### The loss function to estimate the value of variable-rate application

Based on the differences in ${\text{E}}\left[L\left(F\right)\right]$ between ${F}_{\text{opt}}$ and ${F}_{\text{UA}}$, there appears to be little financial incentive for variable-rate application of K fertiliser in Field 4 ($\Delta {\text{E}}\left[L\left(F\right)\right]=1$, Table 3). The difference in ${\text{E}}\left[L\left(F\right)\right]$ between ${F}_{\text{opt}}$ and ${F}_{\text{UA}}$ is larger for Fields 1, 2 and 3 (with $\Delta {\text{E}}\left[L\left(F\right)\right]$ equal to 7, 6 and 81, respectively). For available P, most kriged estimates lay in the linearly increasing range of the dose–response curve, and there is a financial incentive to implement variable rate application for all fields.

The difference in total K fertiliser used between ${F}_{0}$ and ${F}_{\text{opt}}$ was especially large for Fields 1, 2 and 3, which can be explained by the large nugget variance (${c}_{0}$) and sill (${c}_{1}$) (Fig. 1 and Table 2). Field 4 has the largest ${\text{E}}\left[L\left(F\right)\right]$ values for P under VRA (Table 3), which can be attributed to large values of ${\sigma }_{\text{krig}}^{2}$ (Fig. 7), a short distance parameter ($a$) and large sill (${c}_{1}$) in the variogram (Fig. 1 and Table 2). Additionally, the smaller applications of P fertiliser under VRA than under uniform application for Fields 1, 2 and 4 means that VRA poses less environmental damage than uniform application would; that is, there would be less P lost from the soil to pollute waterways and cause eutrophication.

The large expected loss under uniform application of P in Fields 3 and 4 can be attributed to a biased estimate of the mean concentration of soil P across the field and hence blanket fertilizer recommendation, ${F}_{\text{UA}}$. Sampling by a W-design has been found to be equivalent to random sampling in the estimation of a mean concentrations of nutrients (Marchant et al., 2012). However, because the samples that make up the W-design were chosen a posteriori (the samples come from the set that were analysed by wet chemistry and these were selected purposely to span the range in the field) the mean estimate of available P and K is suspected to be biased.

It is further noted that the expected loss under uniform application of K fertiliser is large for Field 3. The large expected loss is likely due to the effect of uncertainty being more pronounced for smaller values of available K (Fig. 4D–F). Additionally, the uncertainty in available K predictions was relatively larger than the range of available K in the calibration set (Supplementary Fig. 2).

Overall, the expected loss, ${\text{E}}\left[L\left(F\right)\right]$, and hence ${F}_{\text{opt}}$ was found to depend on (a) the kriging variance, (b) the ranges of P and K for which the dose–response curves were calibrated, (c) the range of estimated values in the fields and (d) the asymmetry of the loss function. These factors need to be properly quantified, parameterized and accounted for in the loss functions so that farmers can make their decisions with confidence, while taking into account uncertainty.

### Implications of the loss function approach on decision-making

Although quantification of uncertainty (based on data and current models) allows one to make statements with confidence, it can also identify where the effort of reducing uncertainty will result in the largest gains. The loss function has enabled scientists and managers to decide how much field-work and analysis is required to answer specific questions in environmental monitoring. For example, it is used to optimise the size of samples for survey; see Yates (1981), Faechner et al. (2000), Goovaerts (1997), Lark and Knights (2015) for examples. Relevant decisions within a sampling campaign involve (a) where and when to take samples, (b) what measurements to make on the samples, and (c) with what accuracy to take these measurements.

The loss function framework provides a method to assess the quality of predictions from spectroscopy beyond specific metrics such as *R*^2^ and investigate whether the accuracy is “sufficient” to address relevant questions. In this particular study to test the hypothesis whether soil spectroscopy could adequately predict the spatial variability to justify variable rate application of P and K fertiliser.

For example, if sampling, handling and spectroscopy costs are less than the difference between ${\text{E}}\left[L\left({F}_{\text{opt}}\right)\right]$ and ${\text{E}}\left[L\left({F}_{\text{UA}}\right)\right]$ then VRA is worthwhile. These costs depend amongst other things on both the total number of samples and the number of samples for calibration. The costs of spectroscopy per sample will also decrease with increased size of field. The smallest estimate of cost was for Field 2 (the largest in area); it was £49 ha^−1^ for P and £47 ha^−1^ for K. Although these costs fall within the range of cost savings of VRA compared with UA (£7–121 ha^−1^), they are larger than the savings for Field 2 specifically. However, this study investigated a best-case scenario for spectroscopy and did not optimize the data-collection for cost-effectiveness. One could reduce these costs by measuring the reflectance spectra of the soil surface on the run in the field. So far, however, trials to estimate P and K in the soil from visible–NIR in the field by Cozzolino et al. (2013), Daniel et al. (2003), Kuang et al. (2012) and Ji et al. (2014) have produced disappointing results. Reports of *R*^2^ values lie in the range 0.09–0.87 for predictions of available P and 0.03–0.87 for available K. No reports were found that support within-field estimation of available P and K from MIR and XRF spectroscopy, which holds potential for further research.

Within six out of eight fields in this study, the cost-effectiveness of VRA was primarily driven by increases in yield, in some cases at the cost of applying more fertiliser compared to uniform application. The excess use of potash fertiliser does not pose a direct threat to the environment. The overuse of phosphate fertiliser on the other hand in Field 3, might minimise economic loss at the cost of the environment. However, one could argue that increased efficiency gained by VRA also has potential to reduce the total land area used to reach equivalent yields. Setting aside agricultural land in areas vulnerable to leaching is a recognized strategy to manage phosphorus concentrations at the catchment scale (Schoumans et al., 2014). In that case one would need to ensure that leaching or artificial drainage is not the driving force behind small concentrations of P at the field-scale in the first place (Baveye & Laba, 2015). The results further showed that the environmental benefit of fertiliser savings from VRA (in the range of 4–17 kg ha^−1^) was not strictly accompanied with a large increase in profit (Field 1). Hence, in order to account for the environmental benefits of precise fertiliser application, the results suggest that costs of P leaching (e.g. remediation) need to be included in sustainable phosphorus management strategies aimed at precise fertiliser application. These costs of P leaching can be substantial. For example, if the excess fertiliser finds its way into water bodies, the cost to water companies has been estimated to range between 75 and 114 million pounds sterling per year for England and Wales (Pretty et al., 2003). Costs associated with eutrophication range among others from restorative measures such as dredging, treatment of drinking water (including removal of algal toxins), loss of important species and ecological damage generally (Pretty et al., 2003). The presented framework for estimating financial losses could be adapted to place a larger penalty on over-application of phosphorus fertiliser based on the environmental costs of leaching. It could provide a stepping stone towards fulfilling the requirement to quantify the economic and environmental benefits of sustainable phosphorus management (Brownlie et al., 2021).

## Conclusions

The results show that there was an economic incentive for precise fertiliser application of both phosphorus and potassium fertiliser once the uncertainty in soil's nutrient concentrations estimated from sensors was accounted for. Given that growers need to subtract the costs of sampling and sample analysis from their gross income, further study should use the loss function to define an optimum where both uncertainty of information and the effort to collect the data by sampling and analysis are minimised.

To quantify society's benefits of precise fertiliser application holistically, however, environmental costs need to be taken into consideration. The results showed that environmental benefits occurred from precise fertiliser application even though no large increase in profit was gained. These findings have implications for policies aimed at sustainable management of phosphorus fertilisers. That is, it is recommended that the loss function could be adapted to include environmental costs of P leaching to assist in quantifying both the economic and environmental benefits of precise fertiliser application.

## Supplementary Information

Below is the link to the electronic supplementary material.

Supplementary file1 (PDF 2217 kb)
