# Supplementary material for: A loss function to evaluate agricultural decision-making under uncertainty: a case study of soil spectroscopy
Source: Precis Agric. 2022 Mar 12;23(4):1333–53. doi: 10.1007/s11119-022-09887-2 (PMC9239958; doi:10.1007/s11119-022-09887-2)
Supplement: Supplementary file 1 — Supplementary file1 (PDF 2217 kb) [file 11119_2022_9887_MOESM1_ESM.pdf]

Supplementary material

A loss function to evaluate agricultural decision-making under uncertainty: a case study of soil spectroscopy

T.S. Breure[1,2] · **S.M. Haefele**[2] · **J.A. Hannam**[1] · **R. Corstanje**[1] · **R. Webster**[2] · **S. Moreno-Rojas**[3] · **A.E. Milne**[2]

Corresponding author: T.S. Breure

timo.breure@rothamsted.ac.uk

[1] Cranfield University, Cranfield, Bedfordshire MK43 0AL, Great Britain

[2] Rothamsted Research, Harpenden AL5 2JQ, Great Britain

[3] Gs Growers Ltd, Ely CB7 5TZ, Great Britain

The case-study area and the soil sampling campaign

In line with the manuscript, Supplementary Figure 1 shows the field boundaries and the sampling locations for each sampling design.

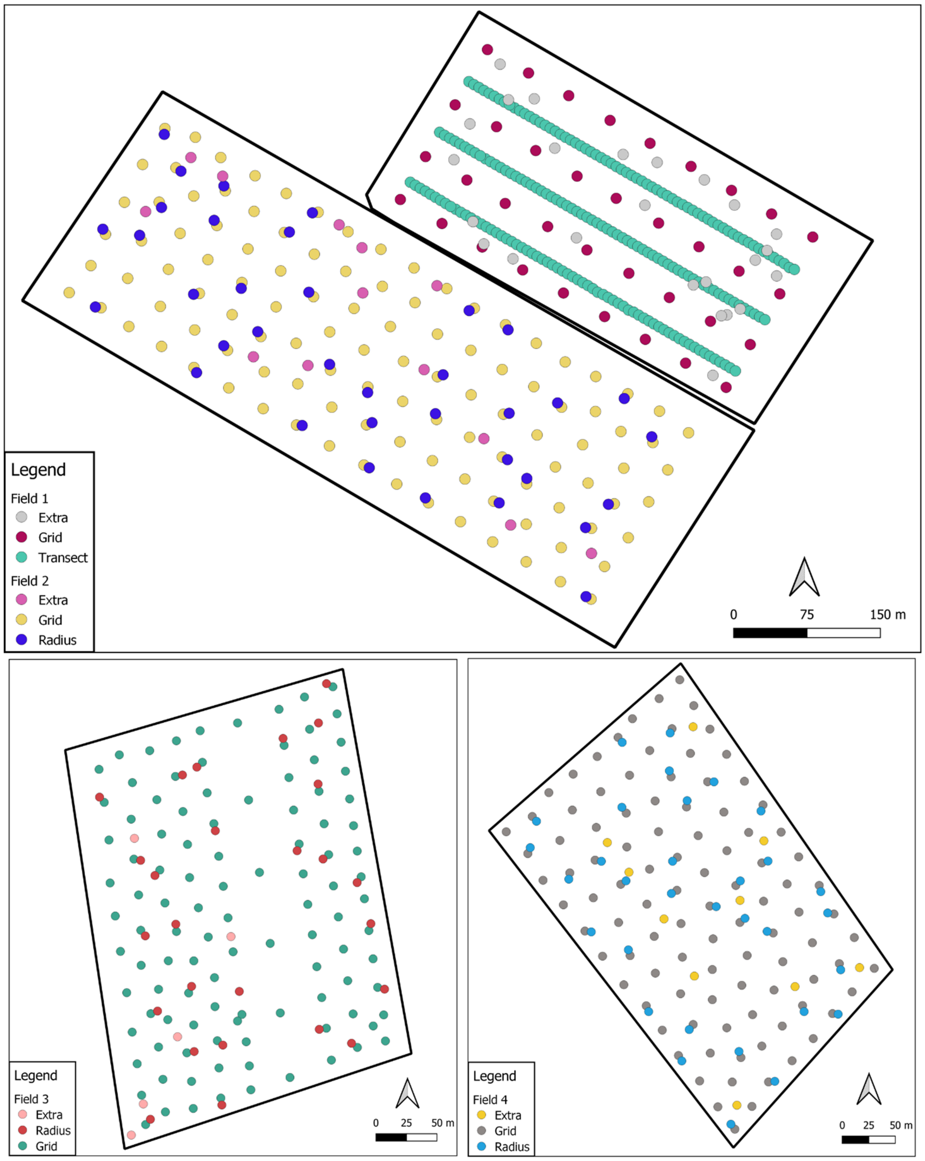

Figure 1: Field boundaries and the sampling locations for each sampling design.

Methods and results of calibration regression

This section gives the methods and results of the calibration regressions computed through partial least squares (PLS) methods and Granger-Ramanathan model-averaging. (See below, Supplementary Figure 2).

The partial least squares regressions were computed for the datasets of Field 1 and 2 combined (given their close proximity), and for Field 3 and 4 separately.

Derivation of the profit function

When we write out the profit equation in its full form;

$$\Phi(F) \;=\; M \times \alpha + \eta R^{\xi F + S} + \nu(\xi F + S) - V \times F \;, \tag{1}$$

its derivative is given by;

$$\Phi' \;=\; M(\eta R^{F\xi + S} \xi ln(R) + \nu\xi) - V \tag{2}$$

we then solve the derivative for 0, given by;

$$\Phi'(0) \;=\; \frac{-S ln(R) + ln(-\frac{-\nu\xi M - V}{ln(R)\eta\xi M})}{\xi ln(R)} \tag{3}$$

which simplifies to Equation 8 in the main text;

$$F_0 \;=\; \ln\left(\frac{B/\xi - \nu}{\eta R^S \ln R}\right)/\xi \;\; \ln R \;, \tag{4}$$

Calculations to derive the increase in available P per kg phosphate fertiliser applied

We provide an example for phosphorus:

Given the support of our kriging predictions; a block of 2m×2m×0.25m soil weighs 480 kg, given a bulk density value of 480 kg m^{-3} (Milne et al. 2006).

Based on Muhammed et al. (2017) 0.18 kg^{-1} / ha^{-1} becomes available to the crop, which is equivalent to adding 0.18 kg^{-1} / 10000m^2.

Which in turn is the equivalent to adding 0.000018 kg^{-1} / m^2 so 0.000018×4 / 2m×2m = 0.000072 / 2m×2m = 72mg to 2m×2m.

Hence 72 mg of P added to 480 kg^{-1} of soil = 0.15 mg / kg^{-1}.

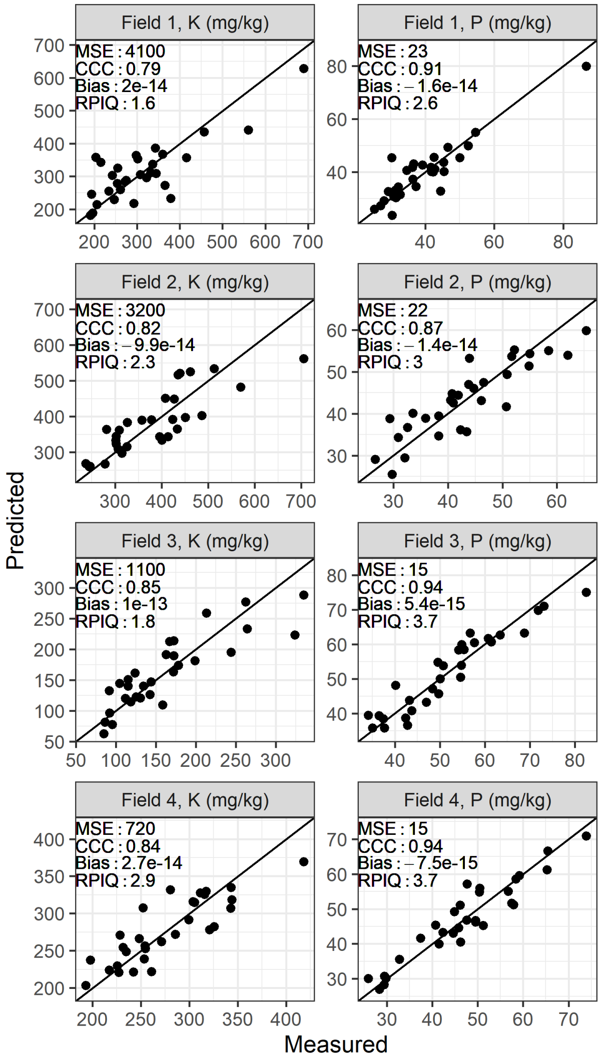

Figure 2: Predicted versus measured of available K (mg kg^{-1}) and available P (mg kg^{-1}) from the Granger-Ramanathan model-average of NIR, MIR and XRF partial least squares predictions. MSE refers to the mean-squared error, CCC the Lin's concordance correlation coefficient (Lin, 1989) and RPIQ the ratio of performance to inter-quantile range (Bellon-Maurel et al. 2011).

Leave-one-out cross-validation of mixed model variograms

The linear mixed model variograms were cross-validated by leave-one-out cross-validation (LOOCV)as implemented in the R package **geoR**. The linear mixed model was re-estimated for each iteration to diminish bias in parameter values (Hastie et al., 2009, Section 7.10).

Leave-one-out cross-validation of the linear mixed-models were evaluated with the mean- and median-standardized squared prediction error (SSPE). The mean SSPE indicates whether the overall error is biased towards being to large or too small. In an ideal case, mean SSPE $\equiv 1.0$. Smaller values indicate that the kriging prediction standard errors overestimate the actual prediction error. Values greater than 1.0 indicate that the standard error on the kriging predictions underestimate the actual prediction error. The median SSPE gives an indication on how the kriged estimates approximate the random variable's variance. If the median SSPE < 0.455 then the kriging overestimates the variance; if it exceeds 0.455 then the kriging underestimates the actual variance of the random variable (Lark, 2000).

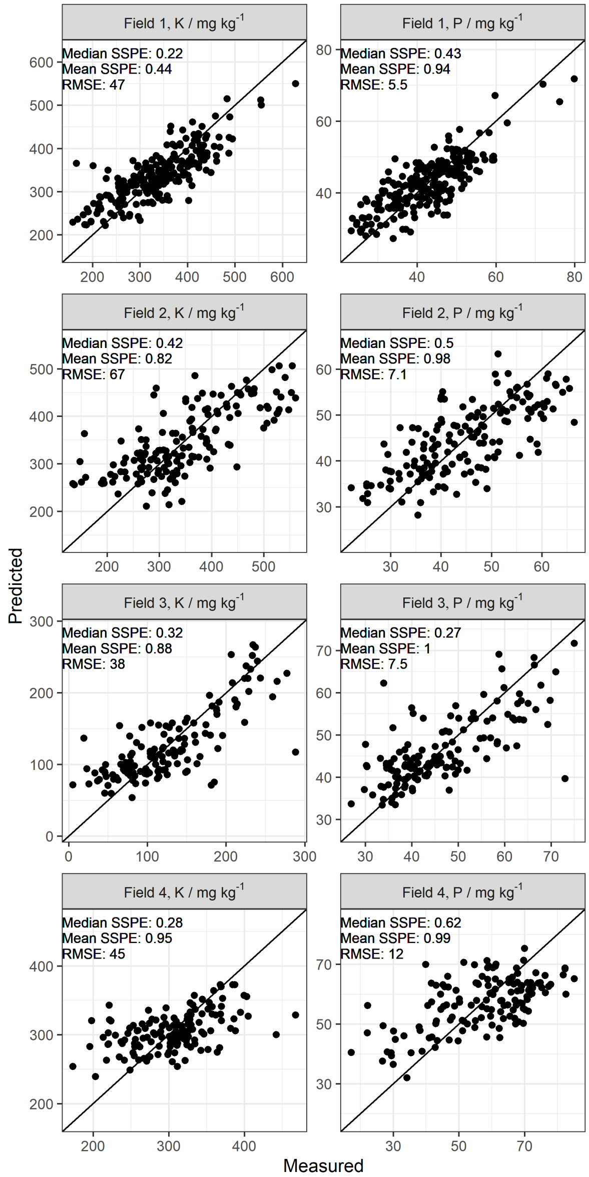

Figure 3: Leave-one-out cross-validation predictions from the linear mixed model variograms. SSPE refers to the standardised squared prediction error, RMSE stands for the root mean squared error.
